# Supplementary material for: The quantitative metabolome is shaped by abiotic constraints
Source: Nat Commun. 2021 May 26;12:3178. doi: 10.1038/s41467-021-23214-9 (PMC8155068; doi:10.1038/s41467-021-23214-9)
Supplement: Supplementary file 1 — Supplementary Information [file 41467_2021_23214_MOESM1_ESM.pdf]

The quantitative metabolome is shaped by abiotic constraints

Amir Akbari[1], James T. Yurkovich[1,2], Daniel C. Zielinski[1], Bernhard O. Palsson[1,3]

[1] *Department of Bioengineering, University of California San Diego, La Jolla, CA 92093*
[2] *Institute for Systems Biology, Seattle, WA 98109*
[3] *Novo Nordisk Foundation Center for Biosustainability, Technical University of Denmark, 2800 Lyngby, Denmark*

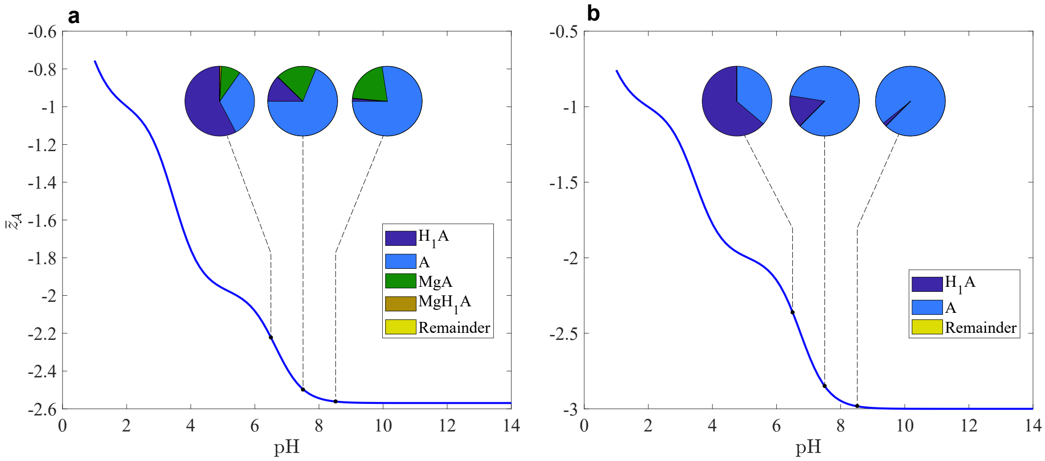

Figure S1: Effective charge of D-glycerate 2-phosphate, related to Fig. 1. (a) $I = 300$ mM and pMg = 2.699. (b) $I = 300$ mM and pMg = 16. Pie charts indicate the distribution of dominant species at the respective pH.

Email addresses: `amakbari@ucsd.edu` (Amir Akbari), `palsson@ucsd.edu` (Bernhard O. Palsson)

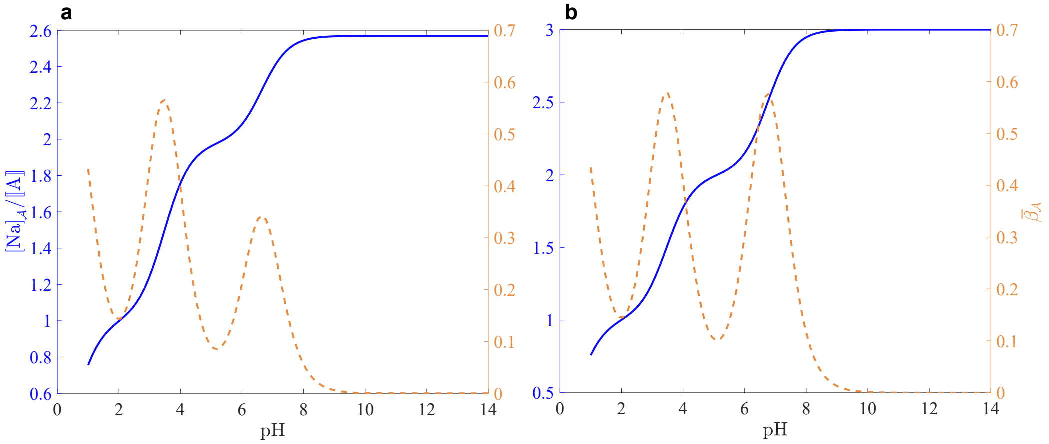

Figure S2: Strong-base equivalent (solid) and buffer intensity (dashed) of D-glycerate 2-phosphate, related to Fig. 1. (a) $I = 300$ mM and pMg $= 2.699$. (b) $I = 300$ mM and pMg $= 16$.

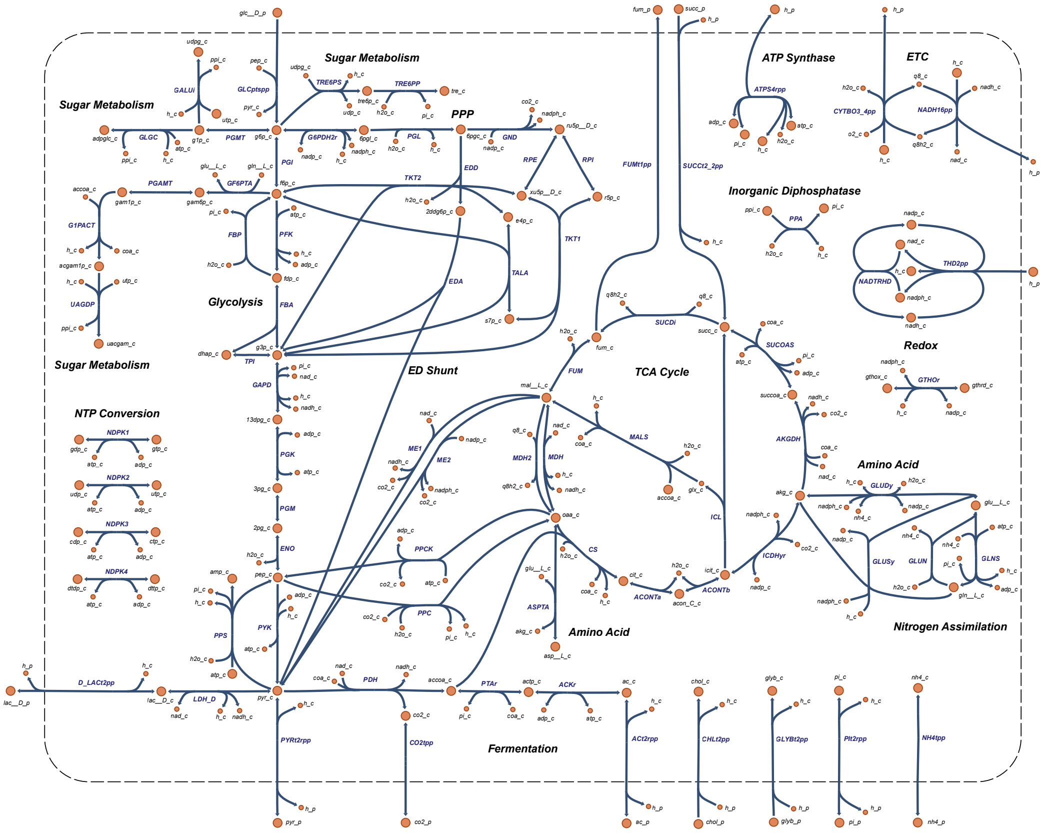

Figure S3: Metabolism in *E. coli*. A reduced metabolic network, comprising more than 90% of the entire metabolome by mole, is constructed to simulate growth on four carbon sources, including glucose, acetate, pyruvate, and succinate.

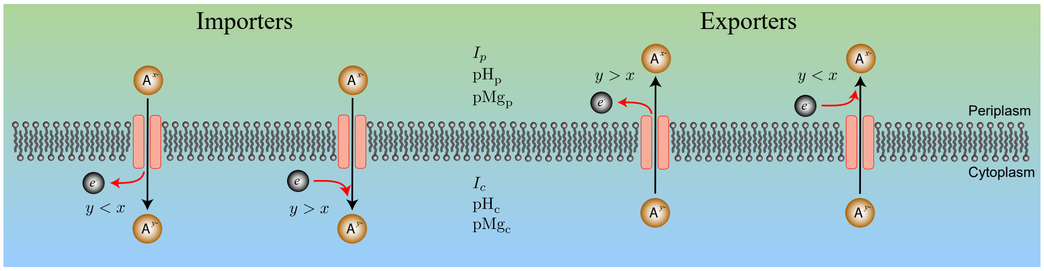

Figure S4: Convention adopted to calculate the reaction charge consumption of transporters, related to Fig. 1. Charge exchange between reactants and water is assumed to take place on the product side of transport reactions.

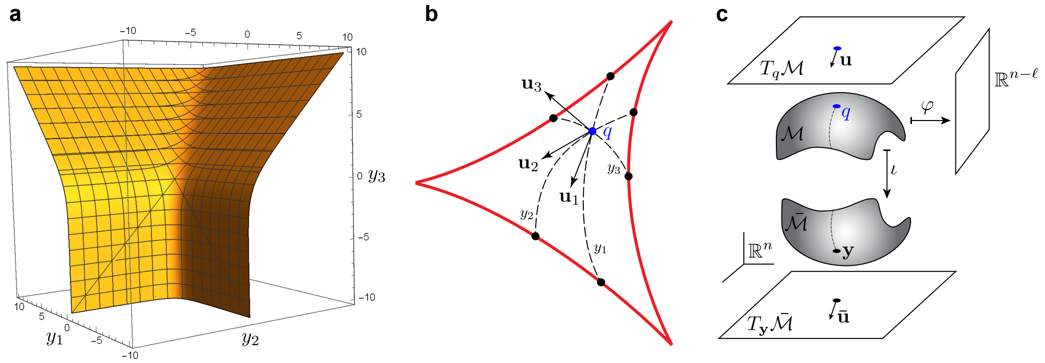

Figure S5: Geometry of the CSS in the Y space, related to Fig. 2. (a) A two-dimensional example of equality-constraint manifold $\bar{\mathcal{M}}$ embedded in $\mathbb{R}^3$, where $\mathbf{A} = [1\ 1\ -1]$ and $f^{\mathrm{eq}} = 1$. (b) Schematic representation of the trajectory-tracing method used to characterize the CSS. Trajectories are constructed in $\bar{\mathcal{M}}$ from an interior point $\mathbf{y}$ along the vectors $\bar{\mathbf{u}}_1$, $\bar{\mathbf{u}}_2$, and $\bar{\mathbf{u}}_3$ that are randomly generated in the tangent space $T_{\mathbf{y}}\bar{\mathcal{M}}$ and continued until they cross at least one of the thermodynamic constraints (red curves). The expectation and standard deviation of any function defined on $\mathcal{C}$ are ascertained by computing the respective line integrals along these trajectories. (c) Schematic representation of the equality-constraint manifold $\mathcal{M}$, tangent space at q, and their embeddings in $\mathbb{R}^n$.

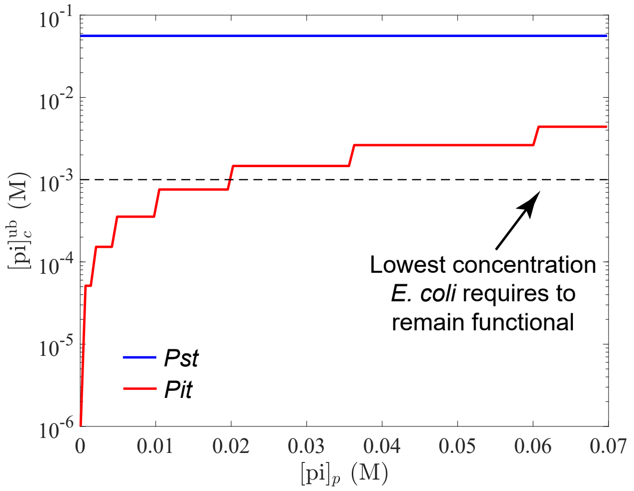

Figure S6: Comparison of the upper bound on the intracellular phosphate concentration in mutants, where either the *Pit* or *Pst* system is active, related to Fig. 3. The upper bounds are plotted as functions of the periplasmic phosphate concentration $[\![\mathrm{pi}]\!]_p$.

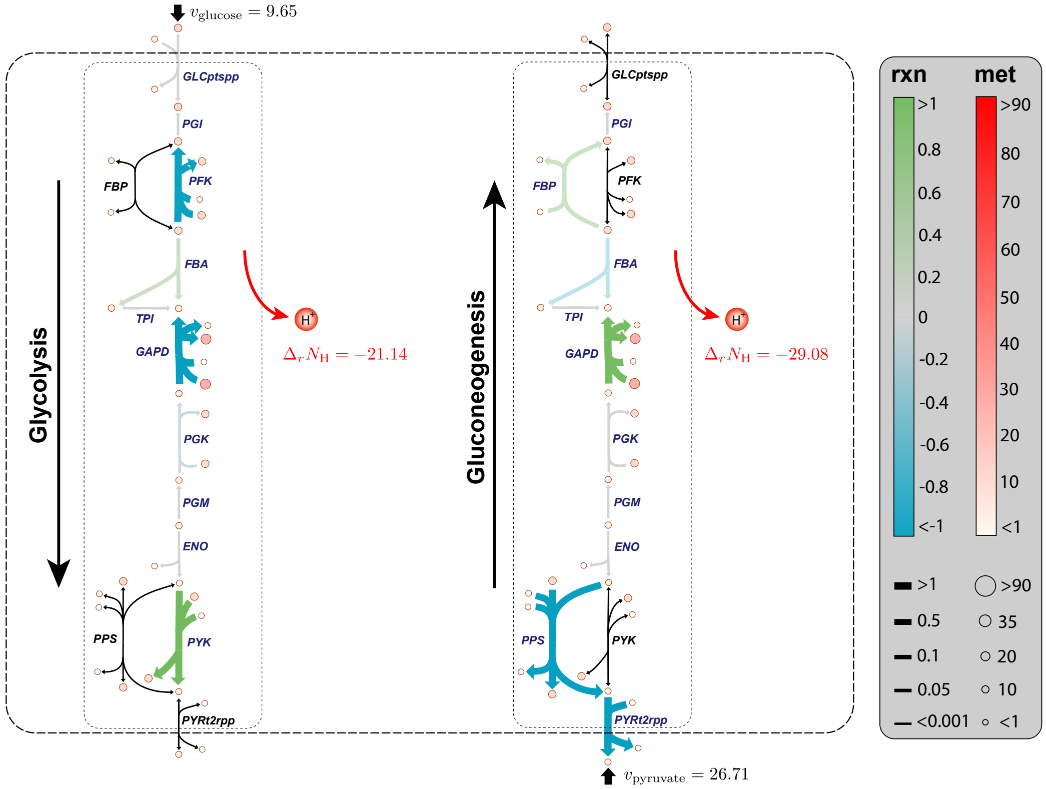

Figure S7: Contribution of glycolytic and gluconeogenetic reactions to pH homeostasis, related to Fig. 5. Carbon-source uptake rate v and pathway hydrogen consumption $\Delta_r N_{\mathrm{H}}$ shown in the figure are measured in mmol/gDW/h and mmol-H/gDW/h, respectively. The uptake rate of carbon sources are determined from the experimental flux measurements of Gerosa *et al.* [14]. Reaction colorbar (rxn) indicates intrinsic hydrogen consumption in mol-H/mol-rxn and metabolite colorbar (met) indicates metabolite hydrogen content in mol-H/mol-met. Arrows point in the direction, in which hydrogen ion is consumed. Unlike Fig. 4c, reaction hydrogen consumption for transport reactions, as indicated by the colorbars, accounts for translocated hydrogen ions. Reactions represented by thin black arrows are inactive.

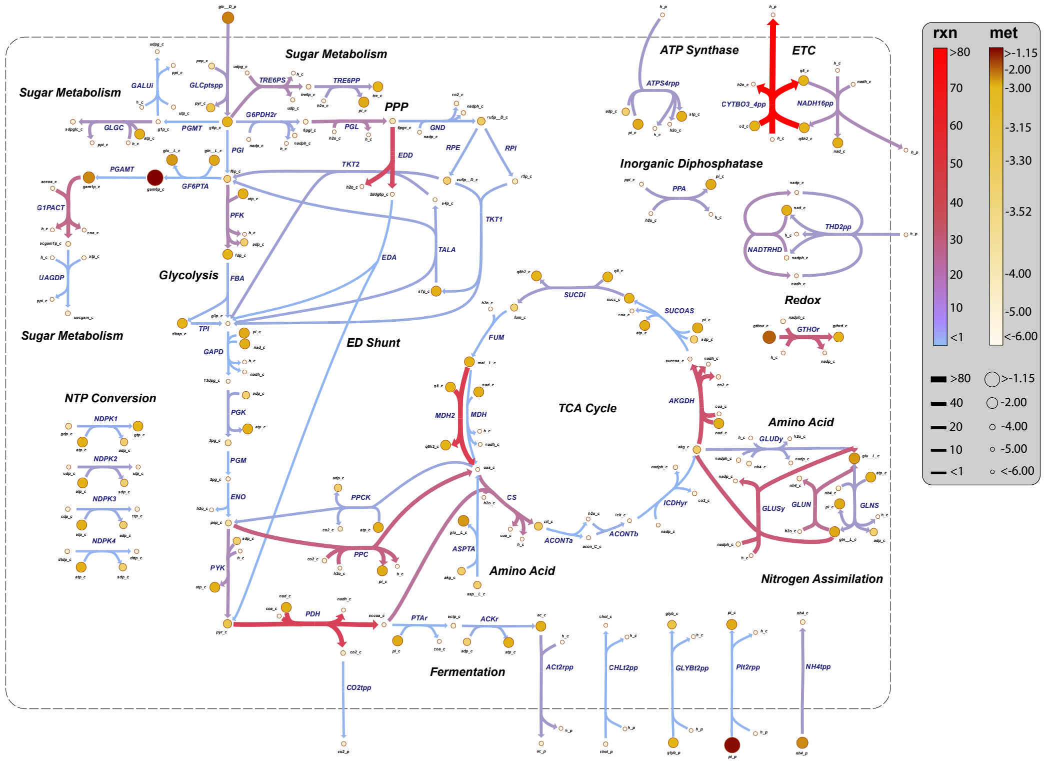

Figure S8: Energy and concentration map, related to Fig. 4. The description of the legend is identical to Fig. 4a.

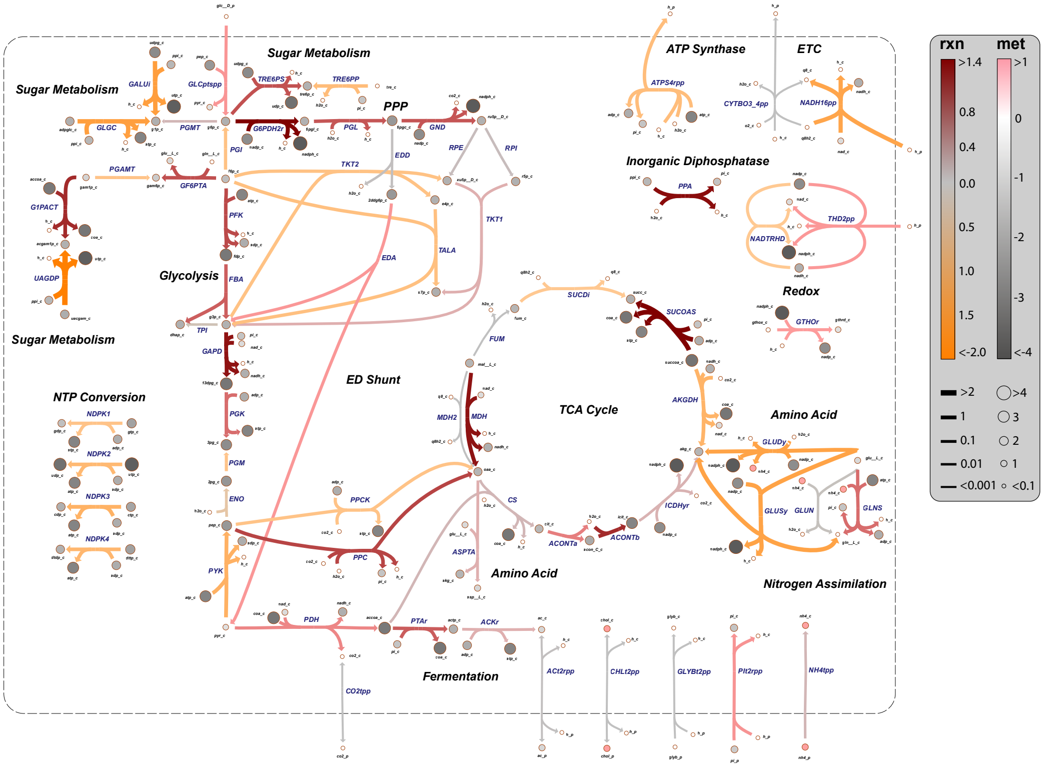

Figure S9: Charge map, related to Fig. 4. The description of the legend is identical to Fig. 4b.

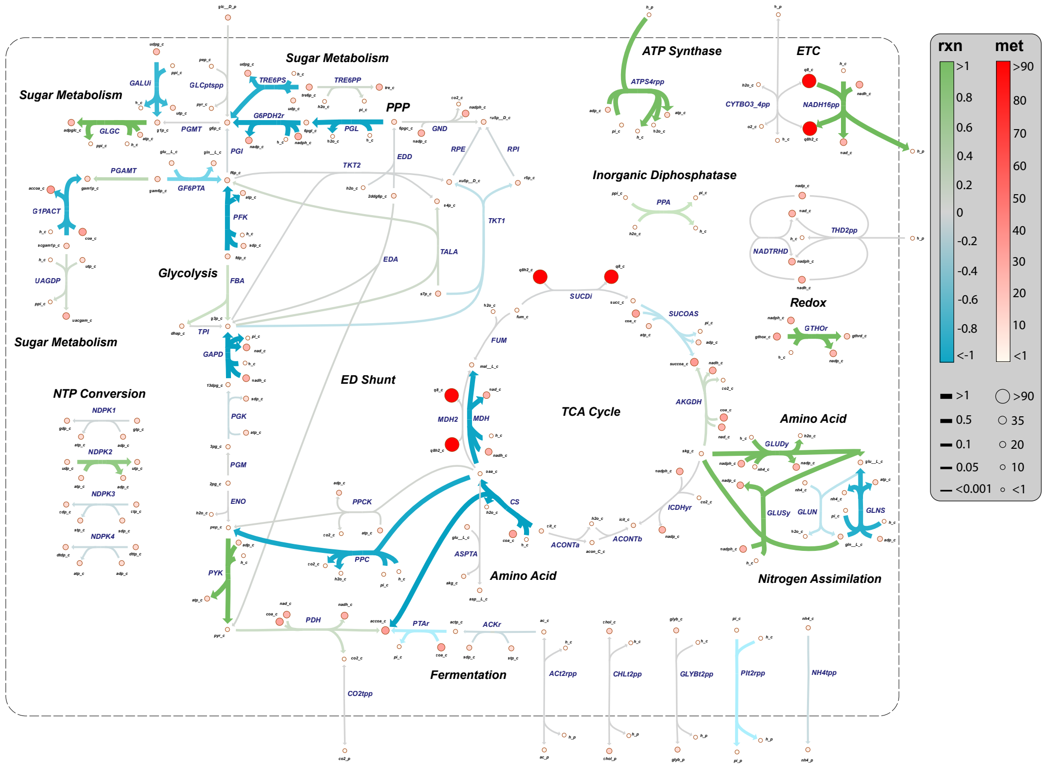

Figure S10: Hydrogen map, related to Fig. 4. The description of the legend is identical to Fig. 4c.

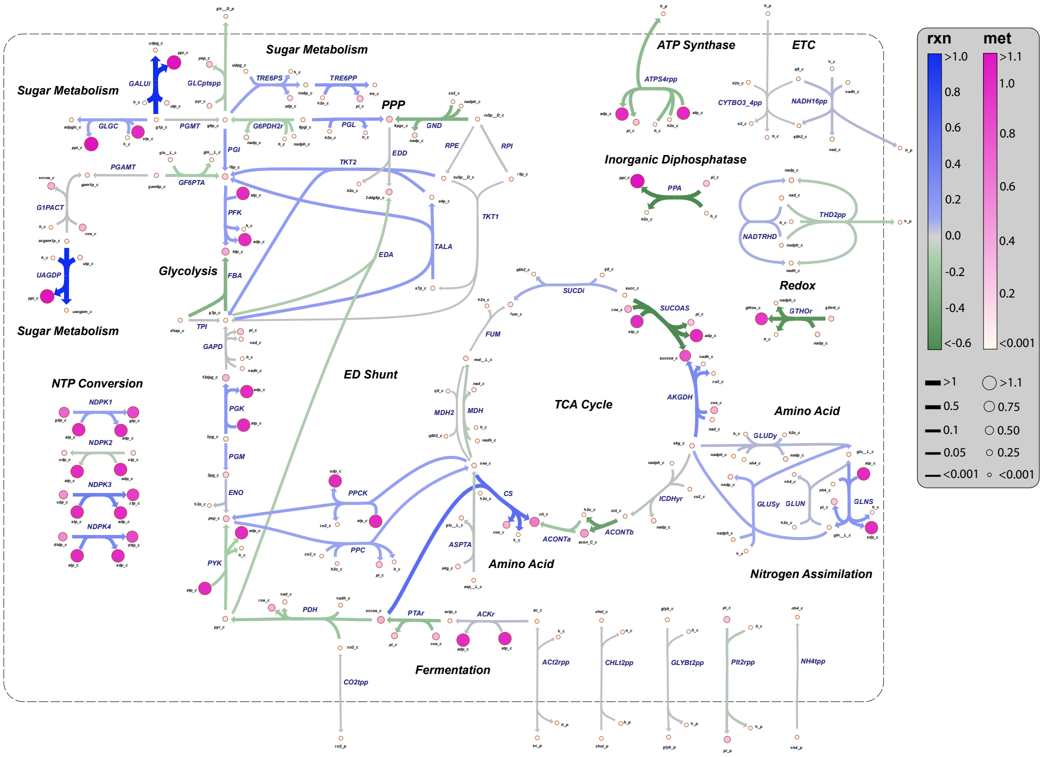

Figure S11: Magnesium map, related to Fig. 4. The description of the legend is identical to Fig. 4d.

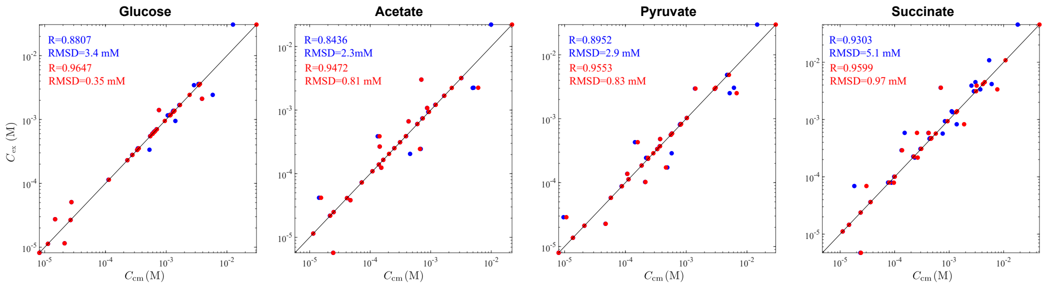

Figure S12: Consistency of the ABCs with metabolomic data, related to Fig. 2. The closest point inside the CSS to measured intracellular concentrations exhibits a higher correlation with metabolomic data when the CSS is defined by thermodynamic constraints alone (red circles) than by all the ABCs (blue circles), highlighting the restrictive characteristics of the charge-related constraints of the ABC-based analysis.

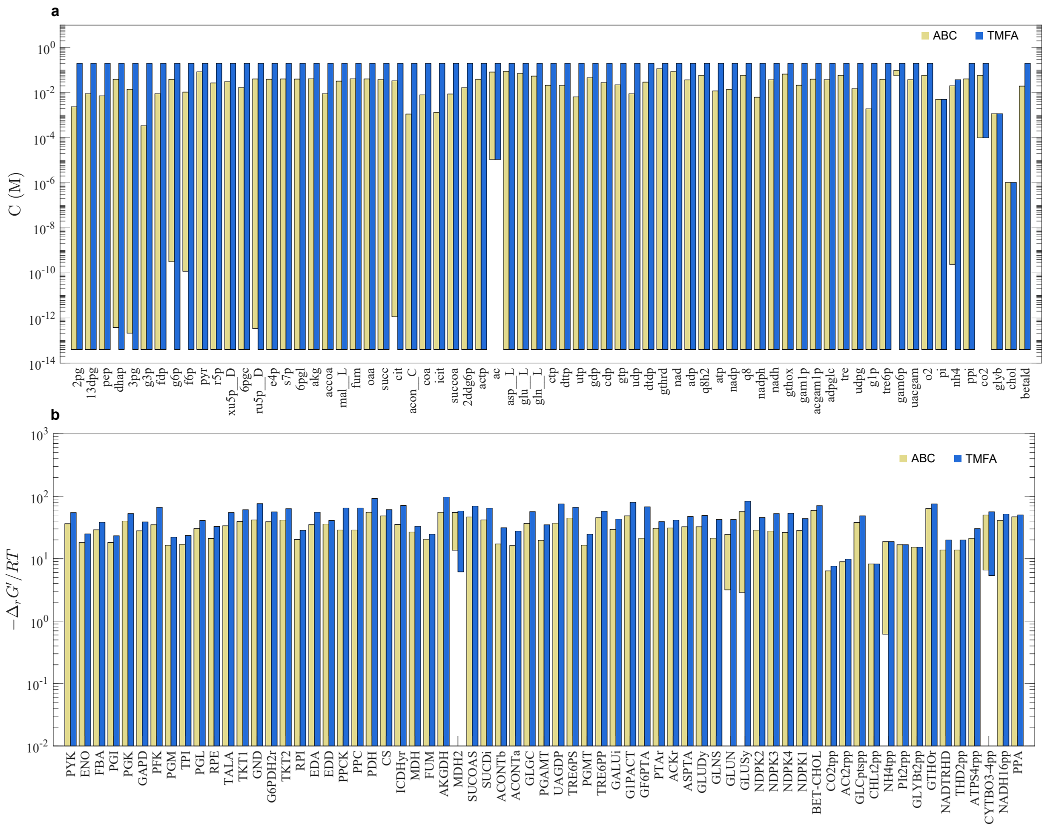

Figure S13: Comparison of the feasible ranges of metabolite concentrations (a) and reaction Gibbs energies (b) furnished by the ABC-based analysis and TMFA when confidence intervals of reaction Gibbs energies are accounted for, related to Fig. 3.

Table S1: Composition of M9 minimal medium, related to Fig. 1.

Compound	Concentration	Dilution factor
Na_2HPO_4	33.9 g/L	0.2
KH_2PO_4	15.0 g/L	0.2
NH_4Cl	5.0 g/L	0.2
NaCl	2.5 g/L	0.2
$CaCl_2$	0.0001 M	1
$MgSO_4$	0.002 M	1
CO_2	0.0001 M	1
Choline	0.0001 M	1
Acetate	0.0001 M	1
Glycine betaine	0.001 M	1
Carbon source	5 mg/L	1

Table S2: Model parameters. Intracellular concentrations of transmembrane ions are approximated using values reported in the literature [59, 60]. These parameters characterize the growth of *E. coli* during the exponential phase, related to Fig. 1.

Parameter	Value	Unit	Parameter	Value	Unit
T	25	°C	I_c	300	mM
$\Delta\Pi$	303	kPa	$\mathcal{B}_c$	62.5	mM
$\Delta\psi$	-140	mV	ζ_c	0	mol-e/L
$\hat{\rho}_w$	1	kg/L	$[\mathrm{K}]_c$	200	mM
pH_p	7.0	–	$[\mathrm{Na}]_c$	14	mM
pH_c	7.5	–	$[\mathrm{Cl}]_c$	20	mM
V_{cell}	2.6	fL	$[\mathrm{Mg}]_c$	2	mM

Table S3: Pathway abbreviations, related to Figs. S3 and 2.

Abbreviation	Pathway
PPP	Pentose phosphate pathway
TCA	Tricarboxylic acid cycle
ED	Entner-Doudoroff Pathway
AA	Amino acid biosynthesis
Fer	Fermentation
NTP	Nucleoside triphosphate conversion
GB	Glycine betaine biosynthesis
ETC	Electron transport chain
ID	Inorganic diphosphatase

Table S4: Relaxation of upper and lower bounds on metabolite concentrations and reaction Gibbs energies furnished by the ABC-based analysis and TMFA when confidence intervals for reaction Gibbs energies are accounted for, related to Fig. 3. Numbers reported for C reflect average values over all the metabolites and those reported for $\Delta_r G'$ reflect average values over all the reactions.

	Reduction in lower bound		Increase in upper bound	
	ABC	TMFA	ABC	TMFA
C (mM)	0	0	0.14	17.52
-$\Delta_r G'$ (kJ/mol)	0.2132	2.2583	0.1312	0.6719
