# Supplementary material for: The quantitative metabolome is shaped by abiotic constraints
Source: Nat Commun. 2021 May 26;12:3178. doi: 10.1038/s41467-021-23214-9 (PMC8155068; doi:10.1038/s41467-021-23214-9)
Supplement: Supplementary file 3 — Description of Additional Supplementary Files [file 41467_2021_23214_MOESM3_ESM.pdf]

Description of Additional Supplementary Files

File Name: Supplementary Data 1
Description: (Model parameters when glucose is the carbon source).

File Name: Supplementary Data 2
Description: (Model parameters when acetate is the carbon source).

File Name: Supplementary Data 3
Description: (Model parameters when pyruvate is the carbon source).

File Name: Supplementary Data 4
Description: (Model parameters when succinate is the carbon source).

File Name: Supplementary Data 5
Description: (Effect of confidence intervals on the flux states determined from Eq. (93)).

File Name: Supplementary Code 1
Description: GAMS and MATLAB codes for characterizing the concentration solution space defined by abiotic constraints.
