# Supplementary material for: The quantitative metabolome is shaped by abiotic constraints
Source: Nat Commun. 2021 May 26;12:3178. doi: 10.1038/s41467-021-23214-9 (PMC8155068; doi:10.1038/s41467-021-23214-9)
Supplement: Supplementary file 9 — Supplementary Code 1 [file 41467_2021_23214_MOESM9_ESM.zip › Codes/Readme.docx]

**Installation guide**

Note: This package requires the General Algebraic Modeling Language (GAMS), a global optimization solver (e.g. BARON), a local nonlinear programming solver (e.g. CONOPT), and a linear programming solver (e.g. ILOG CPLEX).

- Extract codes.zip in your desired working directory.
- Replace “WorkingDirectory” in all the path strings in GAMS and MATLAB codes with your working directory path.
- Add the folder Generic functions to the MATLAB search path.
- Run the following subroutines (in that order) to save parameters and all necessary data in the respective .mat and .xlsx files that are used for main computations, postprocessing, and visualization:
  - BasicParameters() [Preprocessing]
  - Map_pK() [Preprocessing]
  - Correct_pKa() [Preprocessing]
  - CalcFormationEnergy() [Preprocessing]
  - CalcEffProperties() [Preprocessing]
  - StoichiometryOrdCorr() [Preprocessing]
  - ExtractParameters() [Preprocessing]
  - ExtractEC() [Preprocessing]
  - ExtractExpData() [Preprocessing]
  - ExtractKinetics() [Preprocessing]
  - ExternalMedium() [Preprocessing]
  - ReactionEnergy() [Feasibility-nonlinear\Matlab]
- The default folder, in which to run the subroutines listed above and those discussed in the next two sections, is WorkingDirectory\Core.

**GAMS codes**

Run the following GAMS codes [in Feasibility-nonlinear\GAMS] to generate .gdx files [stored in Shared-data\GDX] that are used by the subroutines in the Postprocessing folder. These codes use a parametric formulation, where all the computations outline in the Method section of the paper are parametrized with respect to $\theta=\left( \frac{I_{c}}{C_{s}},\frac{\mathcal{B}}{C_{s}} \right).$

- NonlinearFeas (optional): Given a $\theta$, this subroutine determines whether this point lies in a feasible region of the parameters space and finds/saves a feasible concentration vector inside the CSS.
- NonlinearFeas_ci (optional): The same as NonlinearFeas, but accounts for the confidence intervals of reaction Gibbs energies provided by group-contribution methods.
- NonlinearFeas_SWP (optional): This performs a similar function to NonlinearFeas. However, instead of performing the computations at a fixed $\theta$, it sweeps the 2D parameter space. Note that running this subroutine to completion may take a long time depending on how fine a grid is chosen.
- MetBoundPi_SWP: This subroutine computes the upper/lower bounds on the intracellular concentration of phosphate as a function of its extracellular concentrations. This routine was used to generate the results shown in Fig. S6.
- MetBound: This subroutine computes the global upper/lower bound on metabolite concentrations and saves results in a .gdx file. Run ExtractMetBound() [Postprocessing] to extract its data and save them in a .mat file, which is then used by MATLAB subroutines in the Postprocessing folder.
- MetBound_ci: The same as MetBound, but accounts for the confidence intervals of reaction Gibbs energies provided by group-contribution methods.
- RxnBound: This subroutine computes the global upper/lower bound on reaction Gibbs energies and saves results in a .gdx file. Run ExtractRxnBound() [Postprocessing] to extract its data and save them in a .mat file, which is then used by MATLAB subroutines in the Postprocessing folder.
- RxnBound_ci: The same as RxnBound, but accounts for the confidence intervals of reaction Gibbs energies provided by group-contribution methods.
- NonlinearFeasCheb: This subroutine computes an interior point of the CSS maximally distanced from all the thermodynamic constraints by solving Eq. (85) and saves the result in a .gdx file. Run ExtractNonlinearFeasCheb () [Postprocessing] to extract its data and save them in a .mat file, which is then used by MATLAB subroutines in the Postprocessing folder.
- NonlinearFeasFit: This subroutine computes a point of the CSS that is minimally distanced from the experimental data (Gerosa et al., Cell Systems, 2015) and saves the result in a .gdx file. Run ExtractNonlinearFeasFit () [Postprocessing] to extract its data and save them in a .mat file, which is then used by MATLAB subroutines in the Postprocessing folder.

**Postprocessing**

Run the following MATLAB subroutines [Postprocessing] to visualize the results of computations performed by other GAMS and MATLAB subroutines:

- ProjOrth() [Feasibility-nonlinear\Matlab]: This subroutine generates random trajectories from the point computed by the NonlinearFeasCheb subroutine discussed above in the CSS to compute the expectations and standard deviations of concentrations and reaction energies. The results of this subroutine is necessary for the computations performed by MeanVar() and RxnViol(). To avoid memory overflow, trajectories are generated in several sets, the data of which are stored in separate .mat files. The number of sets of trajectories is specified by n_set, and the number of trajectories per set if specified by n_dir in this subroutine. Note that, the same values for n_set and n_dir must be used in MeanVar() and RxnViol().
- MeanVar() [Postprocessing]: This subroutine computes the expectations and standard deviations of metabolite concentrations and reaction energies from the trajectory data generated by ProjOrth().
- RxnViol() [Postprocessing]: This subroutine computes the violation probabilities of thermodynamic constraints from the trajectory data generated by ProjOrth().
- PlotMeanVar() [Postprocessing]: This subroutine generates a plot, visualizing feasible concentration ranges, concentration expectations, measured concentrations, and other related graphs, that were used to prepare Fig. 2.
- PlotRxnViol() [Postprocessing]: This subroutine generates a plot, visualizing feasible ranges of reaction energies, violation probabilities of thermodynamic constraints, reaction energy expectations, and other related graphs, that were used to prepare Fig. 2.
- SpeciesDistribution() [Postprocessing]: This subroutine computes the distribution of species and buffer intensity for a given reactant at a given ionic strength, pH, and pMg, and visualizes the results by generating plots such as those shown in Figs. S1 and S2.
- FluxFitToExp() [Flux]: This subroutine computes the flux state of the reduced network by finding a parsimonious solution that best matches experimental data (Gerosa et al., Cell Systems, 2015) by solving Eq. (93).

**Carbon sources**

The carbon source for all the subroutines in the default folder discussed above is glucose. Separate folders for each carbon source have been provided in this package, containing the respective excel files, GAMS codes, and MATLAB codes. To perform computations for other carbon sources, replace the corresponding files in the corresponding subfolder of the default folder (i.e. Core) with these files. Follow the same instructions as those outlined above.
